# Physical Activity Is Associated with a Lower Risk of Osteoporotic Fractures in Osteoporosis: A Longitudinal Study

**DOI:** 10.3390/jpm12030491

**Published:** 2022-03-18

**Authors:** Chan-Yang Min, Jung-Woo Lee, Bong-Cheol Kwon, Mi-Jung Kwon, Ji-Hee Kim, Joo-Hee Kim, Woo-Jin Bang, Hyo-Geun Choi

**Affiliations:** 1 Hallym Data Science Laboratory, Hallym University College of Medicine, Anyang 14066, Korea; joicemin@naver.com; 2 Department of Orthopaedic Surgery, Yonsei University Wonju College of Medicine, Wonju 26426, Korea; berrybearlee@gmail.com; 3 Department of Orthopaedic Surgery, Hallym University College of Medicine, Anyang 14068, Korea; bckwon@hallym.or.kr; 4 Department of Pathology, Hallym Sacred Heart Hospital, Hallym University College of Medicine, Anyang 14068, Korea; mulank@hanmail.net; 5 Department of Neurosurgery, Hallym University College of Medicine, Anyang 14068, Korea; kimjihee.ns@gmail.com; 6 Division of Pulmonary, Allergy, and Critical Care Medicine, Department of Medicine, Hallym Sacred Heart Hospital, Hallym University College of Medicine, Anyang 14068, Korea; luxjhee@gmail.com; 7 Department of Urology, Hallym Sacred Heart Hospital, Hallym University College of Medicine, Anyang 14068, Korea; yybbang@gmail.com; 8 Department of Otorhinolaryngology-Head & Neck Surgery, Hallym University College of Medicine, Anyang 14068, Korea

**Keywords:** physical activity, osteoporosis, osteoporotic fracture, vertebral fracture, hip fracture, distal radius fracture

## Abstract

The purpose of our study was to examine the occurrence of osteoporotic fractures (fxs) according to the level of physical activity (PA) among osteoporosis using the Korean National Health Insurance Service (NHIS) customized database. From NHIS data from 2009 to 2017, osteoporosis was selected as requested. PA was classified into ‘high PA’ (*n* = 58,620), ‘moderate PA’ (*n* = 58,620), and ‘low PA’ (*n* = 58,620) and were matched in a 1:1:1 ratio by gender, age, income within the household unit, and region of residence. A stratified Cox proportional hazard model was used to calculate hazard ratios (HRs) for each type of fx comparing PA groups. The ‘low PA’ group was the reference group. For vertebral fx, the adjusted HR (95% confidence intervals (CIs)) was 0.27 (0.26–0.28) for the ‘high PA’ group and 0.43 (0.42–0.44) for the ‘moderate PA’ group. For hip fx, the adjusted HR (95% CIs) was 0.37 (0.34–0.40) for the ‘high PA’ group and 0.51 (0.47–0.55) for the ‘moderate PA’ group. For distal radius fx, the adjusted HR (95% CIs) was 0.32 (0.30–0.33) for the ‘high PA’ group and 0.46 (0.45–0.48) for the ‘moderate PA’ group. The results of this study suggest that a higher intensity of PA is associated with a lower risk of osteoporotic fxs, including vertebral fx, hip fx, and distal radius fx.

## 1. Introduction

The negative health effects of physical inactivity are already well known [1]. The problem of physical inactivity is expected to worsen due to the novel coronavirus pandemic (COVID-19) [2]. A study from the United States reported that among study subjects, 30% responded that they engaged in less physical activity (PA) during the pandemic [3]. Likewise, among the population of England, the rate of physical activity declined by 30% in 2020 compared to that in the period from 2016 to 2019 [4]. In Korea, the time spent in high-intensity PA decreased in all age and gender groups in 2020 compared to that of 2019 according to the Korean Community Health Survey data [5]. World Health Organization (WHO) reported that lack of physical activity (PA) is one of the risk factors for chronic diseases such as cancers, cardiovascular disease burden, and even death [6]. In other words, PA is crucial to maintain and improve one’s health.

One of the goals of PA is improving osteoporosis, one of the major musculoskeletal diseases [7]. Osteoporosis is characterized by a decrease in bone mineral density (BMD) [8]. Although osteoporosis has no outward symptoms, osteoporotic patients are at critical risk of osteoporotic fractures (fxs), including vertebral fx, hip fx, and distal radius fx [9]. Fxs is a critical concern for older adults and can even lead to death. A previous study from Ontario, Canada reported that the absolute mortality risk within 1 year among participants ≥ 66 years old was 19.5% and 12.5% for men and women with fx, respectively, whereas it was 13.5% and 7.4% for men and women without fx, respectively [10]. Another study from Korea reported that the standardized mortality ratios (SMRs) 2 years after vertebral fracture were 2.53 in men and 1.86 in women compared to the general population [11].

Increasing evidence has demonstrated that PA can increase BMD and lower the risk of osteoporosis, hence reducing the risk of some osteoporotic complications. Several randomized controlled trials (RCTs) evaluated if PA could improve bone strength, including BMD, in osteoporotic postmenopausal women [12]. Specifically, resistance training, impact loading and balance exercise increased total hip BMD [13], maximal strength training, including squat exercise showed higher femoral neck and lumbar spine bone mineral contents (BMC) [14], and aerobic dance improved femoral neck BMD [15]. Moreover, several previous cohort studies confirmed that PA could lower the risk of hip fx in each cohort regardless of osteoporosis [16,17].

However, although several RCT studies demonstrated that PA could increase BMD in osteoporotic patients, few RCT studies have included osteoporotic fx as the primary endpoint because of the limited length of the typical study period. Moreover, in RCT studies, few men were recruited as study participants due to the low frequency of osteoporosis in men. In addition, although several cohort studies have demonstrated that PA could prevent hip fx in the certain cohort, few cohort studies have selected osteoporotic patients as a subject population because of insufficient data. In other words, we could not find a study regarding the association between PA and osteoporotic fx in osteoporosis, although osteoporosis increases the risk of osteoporotic fxs. Moreover, the association between PA and vertebral/distal radius fx was not evident in previous studies.

The purpose of our study was to confirm whether a higher intensity of PA could lower the rate of occurrence of osteoporotic fx at each specific site in osteoporotic men and women. We used the Korean National Health Insurance Service (NHIS) customized database to identify the osteoporotic patients.

## 2. Materials and Methods

### 2.1. Study Population and Participant Selection

Hallym University ethics committee (HALLYM 2019-08-029) approved this study according to the Institutional Review Board (IRB) guidelines.

**Figure 1:**
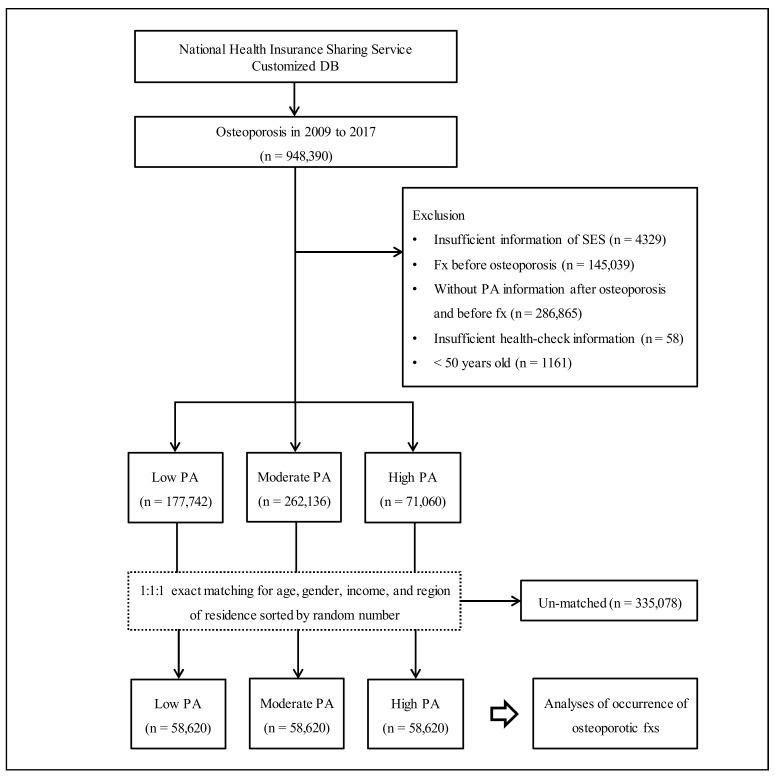
The participant selection flow. Out of a total of 948,390 participants with osteoporosis, ‘high PA’ (*n* = 58,620), ‘moderate PA’ (*n* = 58,620), and ‘low PA’ (*n* = 58,620) were matched by a ratio of 1:1:1 by age, gender, income, and region of residence. Abbreviation: fxs, fractures; PA, physical activity; SES, socioeconomic status.

The Korean National Health Insurance Sharing Service (NHISS) provided the customized database as requested. Among the Korean population who are holding the national health insurance from 2009 to 2017, 948,390 were selected as having osteoporosis according to our definition. Among them, we excluded participants who had insufficient socioeconomic status information (*n* = 4329) or who were diagnosed with osteoporotic fx before osteoporosis diagnosis (*n* = 145,039). Participants were also excluded who had no information on PA after osteoporosis diagnosis and before osteoporotic fx onset (*n* = 286,865). In addition, participants were removed if health-check information was insufficient (*n* = 58) or if they were <50 years old (*n* = 1161). In total, 510,938 participants (*n* = 71,060 with ‘high PA’; *n* = 262,136 with ‘moderate PA’; *n* = 177,742 with ‘low PA’) were included in the study. The ‘high PA’, ‘moderate PA’, and ‘low PA’ groups were matched at a 1:1:1 ratio for gender, age, income within household unit, and region of residence using a random number. The index date was assigned on the day that the PA was first collected before the first diagnosis of osteoporosis. After matching those PA groups, 335,078 participants were removed due to a lack of availability of matched controls. Finally, 58,620 ‘high PA’ subjects, 58,620 ‘moderate PA’ subjects, and 58,620 ‘low PA’ subjects were selected as study participants (Figure 1).

### 2.2. Definition of Osteoporosis (Participants)

We used International Classification of Disease 10th edition (ICD-10) codes and examination insurance claim codes to identify cases of osteoporosis. The definition of osteoporosis cases included participants who were diagnosed or treated with osteoporosis with pathological fx (M80), osteoporosis without pathological fx (M81), or osteoporosis in diseases classified elsewhere (M82) ≥ 2 times and with BMD test using dual energy X-ray absorptiometry (DXA), computed tomography (CT) scans or others [18].

### 2.3. Exposure (Physical Activity)

Information on PA was surveyed according to the International Physical Activity Questionnaire (IPAQ) [19]. The first record of PA information after the first diagnosis of osteoporosis was used. PA groups were defined based on the IPAQ classification. Participants who did vigorous-intensity activity on ≥3 days with ≥1500 metabolic equivalent task (MET)-minutes/week or any combination of moderate- or vigorous-intensity activities or walking ≥7 days with ≥3000 MET-minutes/week were classified in the ‘high PA’ group. Participants who performed moderate-intensity activity or walking ≥5 days with ≥30 min/day, performed vigorous-intensity activity ≥3 days with ≥20 min/day, or any combination of moderate- or vigorous-intensity activities or walking ≥600 MET-minutes/week were classified in the ‘moderate PA’ group. The rest of the participants were classified into the ‘low PA’ group.

### 2.4. Outcome (Time to Event (Osteoporotic Fractures))

The time to event (osteoporotic fxs) was calculated as the month from the date of PA to the censored date or event date. The date of fxs was assigned as the first-time diagnosis of each fx. Osteoporotic fxs were vertebral fx, hip fx, and distal radius fx. The vertebral fx included participants who were diagnosed with fx of thoracic vertebra (S220) or fx of lumbar vertebra (S320) using ICD-10 codes [20]. The hip fx group included participants who were diagnosed with fx of the neck or the femur (S720), pertrochanteric fx (S721), or subtrochanteric fx (S722) using ICD-10 codes [20]. The distal radius fx group included participants who were diagnosed with fx of the lower end of the radius (S525) using ICD-10 codes [21].

### 2.5. Covariates

Age was categorized from 50 years old to ≥85 years old with 5-year intervals (a total of 8 groups). Income within household units and regions of residence were classified based on our previous studies [22,23]. Categories of smoking status, alcohol consumption, and obesity based on body mass index (BMI) were defined as described in our previous studies [22,23]. Blood pressure (BP, including systolic BP (SBP) and diastolic BP (DBP)), fasting blood glucose, and total cholesterol were also collected. The Charlson Comorbidity Index (CCI) score was assigned to each participant to assess the burden of comorbidities [24].

### 2.6. Statistical Analyses

The Kruskal–Wallis test was used to compare the percentage of each characteristic among the PA groups. To compare the cumulative occurrence of each osteoporotic fx among the PA groups, Kaplan–Meier failure analysis and the log-rank test were performed. To analyze the hazard ratios (HRs) with 95% CIs for each osteoporotic fx, including vertebral fx, hip fx, and distal radius fx in the PA groups, a stratified Cox proportional hazard model was used. In this analysis, the crude and adjusted models (adjusted for fasting blood glucose, SBP, DBP, total cholesterol, alcohol consumption, smoking status, obesity, and CCI scores) were fit. The analysis was stratified by gender, age, income within household unit, and region of residence. For the subgroup analyses, age groups (<65 years old and ≥65 years old) and gender (men and women) were recategorized, and the crude and adjusted models were implemented with a stratified Cox model. Other subgroup analyses were performed (Tables S1–S3).

Two-tailed testing was performed, and significance was defined as a *p* value < 0.05. A Bonferroni correction was used to control type 1 errors when calculating the *p* value for three outcomes of osteoporotic fxs (α = 0.05/3). For statistical analyses, SAS Enterprise Guide version 7.13 (SAS Institute Inc., Cary, NC, USA) was used.

## 3. Results

PA groups were exactly matched by a 1:1:1 ratio according to age, gender, income within household unit, and region of residence (all *p* = 1.000). The percentage of obesity, smoking status, alcohol consumption, and CCI score and mean of total cholesterol, BP, and fasting blood glucose were significantly different among the PA groups (all *p* < 0.005). The percentage of subjects with vertebral fx (no. of subjects with vertebral fx/total participants) in the ‘high PA’, ‘moderate PA’, and ‘low PA’ groups was 6.9% (4042/58,620), 10.6% (6233/58,620), and 21.8% (12,787/58,620), respectively (*p* < 0.001). The percentage of subjects with hip fx (no. of subjects with hip fx/total participants) in the ‘high PA’, ‘moderate PA’, and ‘low PA’ groups was 1.2% (687/58,620), 1.7% (984/58,620), and 3.5% (2048/58,620), respectively (*p* < 0.001). The percentage of subjects with distal radius fx (no. of subjects with distal radius fx/total participants) in the ‘high PA’, ‘moderate PA’, and ‘low PA’ groups was 5.1% (2991/58,620), 7.2% (4229/58,620), and 13.9% (8119/58,620), respectively (*p* < 0.001, Table 1).

**Figure 2:**
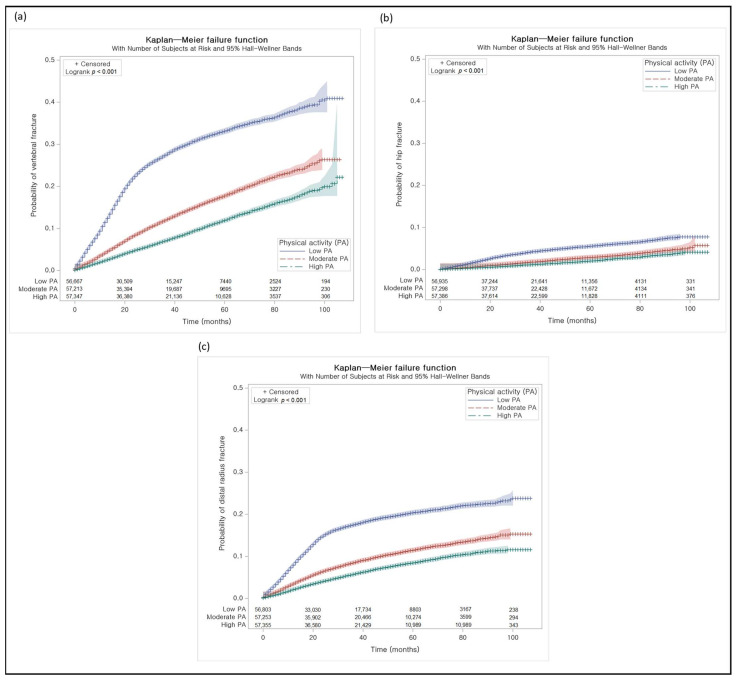
Kaplan–Meier failure analyses. (**a**) The cumulative proportion of vertebral fracture was low in the order of ‘high PA’, ‘moderate PA’, and ‘low PA’. (**b**) The cumulative proportion of hip fracture was low in the order of ‘high PA’, ‘moderate PA’, and ‘low PA’. (**c**) The cumulative proportion of distal radius fracture was low in the order of ‘high PA’, ‘moderate PA’, and ‘low PA’.

Cumulative rate of each fx was higher in order of the ‘low PA’, ‘moderate PA’, and ‘high PA’ groups (log-rank test, each *p* < 0.001, Figure 2).

The adjusted HR (95% CIs) for vertebral fx in the ‘high PA’ group was 0.27 (0.26–0.28) and in the ‘moderate PA’ group was 0.43 (0.42–0.44) as compared to the ‘low PA’ group. In analyses of subgroups defined by age and gender, the findings were consistent with the above findings (Table 2).

The adjusted HR (95% CIs) for hip fx in the ‘high PA’ group was 0.37 (0.34–0.40) and in the ‘moderate PA’ group was 0.51 (0.47–0.55) compared to ‘low PA’. In analyses of subgroups defined by age and gender, the findings were consistent with the above findings in all subgroups (Table 3).

The adjusted HR (95% CIs) for distal radius fx in the ‘high PA’ group was 0.32 (0.30–0.33) and in the ‘moderate PA’ group was 0.46 (0.45–0.48) compared to the ‘low PA’ group. In analyses of subgroups defined by age and gender, the findings were consistent with the above findings in all subgroups (Table 4).

In other subgroup analyses, the findings were also consistent with the above findings in all subgroups for each fx (Supplementary Tables S1–S3).

## 4. Discussion

We confirmed the association between the intensity of PA and the occurrence of each osteoporotic fx in subjects with osteoporosis using the NHIS customized data. Based on our results, the higher the intensity of PA is, the lower the rate of occurrence of each osteoporotic fx. The findings were consistent across all subgroup analyses.

According to previous studies, PA is likely to influence the fx risk. One such study that reviewed randomized controlled trials (RCTs) reported that exercise improved BMD or decreased fall risk to prevent fx risks in postmenopausal women with osteoporosis [12]. Among the RCT studies, one study with 52-week intervention in postmenopausal women with osteoporosis reported that the adjusted mean difference in hip total BMD in the exercise group compared to the control group was 0.012 (95% CI = 0.002 to 0.022, *p* < 0.05) [13]. In another study with a 24-week intervention in postmenopausal women with osteopenia, the change in femoral neck BMD was −1.3 ± 2.7% and 3.1 ± 4.6% in the control group and in the exercise group, respectively (*p* = 0.001) [15]. However, few studies for an association between PA and vertebral fx or distal radius fx in osteoporosis in the RCT study have been performed. Instead, some studies have demonstrated that the type of exercise differently affects BMD in the spine [25,26]. Furthermore, no RCT studies were found with fx as the endpoint.

On the other hand, a meta-analysis study using prospective cohort studies suggested that PA could prevent hip fx (relative risk = 0.62, 95% CI = 0.56–0.69 for women; relative risk = 0.55, 95% CI = 0.44–0.69 for men) [16]. Another meta-analysis of cohort studies also suggested that leisure PA could reduce the risk of hip fx in older women (relative risk = 0.93, 95% CI = 0.91–0.96) [17]. However, no association was found between PA and the risk of distal radius fx or vertebral fx in previous studies. Furthermore, no observational study has used osteoporotic patients as their target study population.

In our study, using the NHIS customized database, we selected osteoporotic men and women who were at risk of osteoporotic fxs, and we examined osteoporotic fxs as the primary endpoint. Due to the large sample size and high statistical power of our study, we found an inverse association between PA intensity and all types of osteoporotic fxs in patients with osteoporosis. Moreover, we found associations according to specific characteristics, such as age and gender due to the large sample size.

Although a high intensity of PA might be considered to be a risk factor for falls and fxs in osteoporosis patients, increasing evidence has demonstrated that increasing PA could prevent fxs, actually reducing falls by resistance and balance training and increased BMD. One review study found that exercise reduced the incidence of one or more fall-related fxs in 10 RCTs (risk ratio = 0.73, 95% CIs = 0.56–0.95). Specifically, the best exercises for reducing falls were balance and functional exercises, such as Tai Chi, and multiple other types of exercise [27]. One of the recommendations of an international panel was that individuals with osteoporosis or osteoporotic vertebral fxs should not perform aerobic PA without resistance and balance PA to prevent falls [28]. Exercises that use body weight or other forms of weight, including resistance training and cycling, are the major reasons why increased PA could lead to higher BMD [29].

We hypothesized that participants might have a higher fx risk if they first started moderate- to high-intensity PA after the onset of osteoporosis. Therefore, additional subgroup analyses were performed for subgroups defined by previous PA intensity before the onset of osteoporosis in subjects for whom previous PA information was available. Surprisingly, the findings were all consistent with the main findings (Supplementary Table S4). Hence, a higher intensity of PA does not seem to be a risk factor for osteoporotic diseases, regardless of whether the participants were not active in PAs prior to the diagnosis of osteoporosis.

Although our study findings show that a higher intensity of PA lowered each osteoporotic fx in osteoporosis, the findings should be cautiously interpreted on the basis of previous studies. Obviously, falls are one of the major risk factors for fxs [9]. In addition, functional impairment is likely to be associated with a high risk of fx [30]. Because data regarding falls or functional impairment was not available in the NHIS customized database, we could not assess the contribution of those factors in our study. Based on the results of previous studies and practical knowledge, the intensity and type of PA should be accounted for depending on the level of functional impairment or risk of falls.

Several limitations of our study mainly regarding using the secondary data should be noted. Some variables were not available, including the history of falls, functional impairment, and dietary intake with supplement intake, including vitamin D and calcium intake. Moreover, in defining the PA groups, the METs were limited because the variable related to time of walking and moderate activity was collected as binary (≥30 min or not), and the variable for vigorous activity was also binary (≥20 min or not). In addition, the association between specific types of PA and fxs in osteoporosis could not be determined from the data. In addition, not only were specific BMD values not available for each participant, but BMD measurements also differed according to the type of instrument in the hospital. We could not confirm whether the actual osteoporotic fx is or not because we defined osteoporotic fxs using only ICD-10 codes. Specific medication regarding protective or affecting bone was not available to adjust in the analysis. Due to the observational study design and based on the above limitations, determination of causality between PA and osteoporotic fxs in osteoporosis should be carefully considered.

The major strength of our study was the use of a national customized database including data from osteoporotic men and women. Because of the large number of subjects, matching by gender, age, income within household unit, and region of residence in a 1:1:1 ratio for each PA group was feasible. Moreover, various lifestyle factors and health indicators, such as alcohol consumption, smoking status, obesity, and CCI scores, were used as covariates. Therefore, the study was uniquely positioned to demonstrate an association between PA and osteoporotic fxs in osteoporosis. In addition, 9 years’ worth of follow-up data were available. Therefore, we had sufficient data to use osteoporotic fxs as endpoints in our study.

## 5. Conclusions

The results suggested that a higher intensity of PA was negatively associated with osteoporotic fxs, including vertebral fx, hip fx, and distal radius fx. In addition, we found that a higher intensity of PA was negatively associated with each osteoporotic fx under various conditions.

## Figures and Tables

**Table 1:** General characteristics of participants.

Characteristics	Total Participants			
	Low PA (*n* = 58,620)	Moderate PA (*n* = 58,620)	High PA (*n* = 58,620)	*p*-Value
Age (years old, *n*, %)				1.000
50–54	4370 (7.5)	4370 (7.5)	4370 (7.5)	
55–59	8419 (14.4)	8419 (14.4)	8419 (14.4)	
60–64	12,257 (20.9)	12,257 (20.9)	12,257 (20.9)	
65–69	11,545 (19.7)	11,545 (19.7)	11,545 (19.7)	
70–74	14,223 (24.3)	14,223 (24.3)	14,223 (24.3)	
75–79	5241 (8.9)	5241 (8.9)	5241 (8.9)	
80–84	2275 (3.9)	2275 (3.9)	2275 (3.9)	
85+	290 (0.5)	290 (0.5)	290 (0.5)	
Gender (*n*, %)				1.000
Men	7046 (12.0)	7046 (12.0)	7046 (12.0)	
Women	51,574 (88.0)	51,574 (88.0)	51,574 (88.0)	
Income within household unit (*n*, %)				1.000
1 (lowest)	11,563 (19.7)	11,563 (19.7)	11,563 (19.7)	
2	10,873 (18.6)	10,873 (18.6)	10,873 (18.6)	
3	13,467 (23.0)	13,467 (23.0)	13,467 (23.0)	
4	12,522 (21.4)	12,522 (21.4)	12,522 (21.4)	
5 (highest)	10,195 (17.4)	10,195 (17.4)	10,195 (17.4)	
Region of residence (*n*, %)				1.000
Urban	25,523 (43.5)	25,523 (43.5)	25,523 (43.5)	
Rural	33,097 (56.5)	33,097 (56.5)	33,097 (56.5)	
Total cholesterol level (mg/dL, mean, SD)	197.9 (44.1)	197.2 (40.1)	196.8 (39.0)	0.002 *
SBP (mmHg, mean, SD)	126.5 (16.0)	125.7 (15.3)	125.6 (15.1)	<0.001 *
DBP (mmHg, mean, SD)	76.5 (9.9)	76.1 (9.6)	75.9 (9.5)	<0.001 *
Fasting blood glucose level (mg/dL, mean, SD)	102.4 (27.3)	100.7 (22.8)	100.6 (22.4)	<0.001 *
Obesity ^1^ (*n*, %)				<0.001 *
Underweight	2433 (4.2)	2022 (3.5)	1526 (2.6)	
Normal	21,127 (36.0)	22,246 (38.0)	22,883 (39.0)	
Overweight	13,857 (23.6)	15,003 (25.6)	15,651 (26.7)	
Obese I	18,274 (31.2)	17,275 (29.5)	16,898 (28.8)	
Obese II	2929 (5.0)	2074 (3.5)	1662 (2.8)	
Smoking status (*n*, %)				<0.001 *
Nonsmoker	52,333 (89.3)	52,950 (90.3)	53,580 (91.4)	
Past smoker	3000 (5.1)	3372 (5.8)	3327 (5.7)	
Current smoker	3287 (5.6)	2298 (3.9)	1713 (2.9)	
Alcohol consumption (*n*, %)				<0.001 *
<1 time a week	51,745 (88.3)	51,022 (87.0)	50,725 (86.5)	
≥1 time a week	6875 (11.7)	7598 (13.0)	7895 (13.5)	
CCI score (n, %)				<0.001 *
0	35,109 (59.9)	38,616 (65.9)	39,930 (68.1)	
1	9807 (16.7)	9107 (15.5)	8552 (14.6)	
≥2	13,704 (23.4)	10,897 (18.6)	10,138 (17.3)	
Osteoporotic fxs (*n*, %)				
Vertebral fx	12,787 (21.8)	6233 (10.6)	4042 (6.9)	<0.001 *
Hip fx	2048 (3.5)	984 (1.7)	687 (1.2)	<0.001 *
Distal radius fx	8119 (13.9)	4229 (7.2)	2991 (5.1)	<0.001 *

CCI, Charlson comorbidity index; DBP, diastolic blood pressure; fx, fracture; PA, physical activity; SBP, systolic blood pressure. * Kruskal–Wallis test. Significance at <0.05 with Bonferroni correction (α = 0.05/3). ^1^ Obesity (BMI, body mass index, kg/m^2^) was categorized as <18.5 (underweight), ≥18.5 to <23 (normal), ≥23 to <25 (overweight), ≥25 to <30 (obese I), and ≥30 (obese II).

**Table 2:** HR (95% CIs) for vertebral fx in the PA groups with subgroup analyses according to age and gender.

Characteristics	No. of Vertebral fx/ No. of Participants	Follow-Up Duration, PY	Incidence Rate, per 100 PY	Hazard Ratios for Vertebral fx				*p* for Interaction
				Crude ^1^	*p*-Value	Adjusted ^1,2^	*p*-Value	
Total participants (*n* = 175,860)								
Low PA	12,787/58,620 (21.8)	108,317	11.8	1		1		
Moderate PA	6233/58,620 (10.6)	129,073	4.8	0.43 (0.41–0.44)	<0.001 *	0.43 (0.42–0.44)	<0.001 *	
High PA	4042/58,620 (6.9)	136,100	3.0	0.27 (0.26–0.28)	<0.001 *	0.27 (0.26–0.28)	<0.001 *	
Age group								<0.001 *
Age < 65 years old (*n* = 38,367)								
Low PA	1962/12,789 (15.3)	26,400	7.4	1		1		
Moderate PA	779/12,789 (6.1)	30,351	2.6	0.35 (0.33–0.37)	<0.001 *	0.36 (0.34–0.38)	<0.001 *	
High PA	450/12,789 (3.5)	31,412	1.4	0.19 (0.18–0.20)	<0.001 *	0.20 (0.18–0.21)	<0.001 *	
Age ≥ 65 years old (*n* = 137,493)								
Low PA	10,825/45,831 (23.6)	81,917	13.2	1		1		
Moderate PA	5454/45,831 (11.9)	98,722	5.5	0.47 (0.45–0.48)	<0.001 *	0.47 (0.45–0.49)	<0.001 *	
High PA	3592/45,831 (7.8)	104,688	3.4	0.31 (0.30–0.32)	<0.001 *	0.31 (0.30–0.32)	<0.001 *	
Gender								0.093
Men (*n* = 21,138)								
Low PA	1917/7046 (27.2)	12,757	15.0	1		1		
Moderate PA	1112/7046 (15.8)	15,246	7.3	0.51 (0.47–0.55)	<0.001 *	0.51 (0.47–0.55)	<0.001 *	
High PA	754/7046 (10.7)	16,382	4.6	0.33 (0.30–0.36)	<0.001 *	0.33 (0.31–0.36)	<0.001 *	
Women (*n* = 154,722)								
Low PA	10,870/51,574 (21.1)	95,560	11.4	1		1		
Moderate PA	5121/51,574 (9.9)	113,827	4.5	0.41 (0.40–0.43)	<0.001 *	0.42 (0.40–0.43)	<0.001 *	
High PA	3288/51,574 (6.4)	119,718	2.7	0.26 (0.25–0.27)	<0.001 *	0.26 (0.25–0.27)	<0.001 *	

CCI, Charlson comorbidity index; CIs, confidence intervals; DBP, diastolic blood pressure; fx, fracture; HR, hazard ratio; PA, physical activity; PY, person–year; SBP, systolic blood pressure. * Stratified Cox proportional hazard model, significance at <0.05 with Bonferroni correction (α = 0.05/3). ^1^ Stratified by gender, age, income within household unit, and region of residence. ^2^ Adjusted for total cholesterol, SBP, DBP, fasting blood glucose, obesity, smoking, alcohol consumption, and CCI score.

**Table 3:** HR (95% CIs) for hip fx in the PA groups with subgroup analyses according to age and gender.

Characteristics	No. of Hip fx/ No. of Participants	Follow-up Duration, PY	Incidence Rate, per 100 PY	Hazard Ratios for Hip fx				*p* for Interaction
				Crude ^1^	*p*-Value	Adjusted ^1,2^	*p*-Value	
Total participants (*n* = 175,860)								
Low PA	2048/58,620 (3.5)	139,819	1.5	1		1		
Moderate PA	984/58,620 (1.7)	142,750	0.7	0.47 (0.44–0.51)	<0.001 *	0.51 (0.47–0.55)	<0.001 *	
High PA	687/58,620 (1.2)	143,707	0.5	0.33 (0.30–0.36)	<0.001 *	0.37 (0.34–0.40)	<0.001 *	
Age group								<0.001 *
Age < 65 years old (*n* = 38,367)								
Low PA	230/12,789 (1.8)	31,829	0.7	1		1		
Moderate PA	81/12,789 (0.6)	32,213	0.3	0.33 (0.28–0.39)	<0.001 *	0.35 (0.30–0.42)	<0.001 *	
High PA	53/12,789 (0.4)	32,338	0.2	0.20 (0.17–0.25)	<0.001 *	0.23 (0.18–0.28)	<0.001 *	
Age ≥ 65 years old (*n* = 137,493)								
Low PA	1818/45,831 (4.0)	107,990	1.7	1		1		
Moderate PA	903/45,831 (2.0)	110,537	0.8	0.52 (0.47–0.56)	<0.001 *	0.56 (0.51–0.61)	<0.001 *	
High PA	634/45,831 (1.4)	111,369	0.6	0.37 (0.34–0.41)	<0.001 *	0.42 (0.38–0.46)	<0.001 *	
Gender								0.009 *
Men (*n* = 21,138)								
Low PA	554/7046 (7.9)	16,666	3.3	1		1		
Moderate PA	311/7046 (4.4)	17,311	1.8	0.54 (0.47–0.63)	<0.001 *	0.59 (0.51–0.68)	<0.001 *	
High PA	199/7046 (2.8)	17,630	1.1	0.35 (0.30–0.41)	<0.001 *	0.40 (0.34–0.47)	<0.001 *	
Women (*n* = 154,722)								
Low PA	1494/51,574 (2.9)	123,153	1.2	1		1		
Moderate PA	673/51,574 (1.3)	125,439	0.5	0.44 (0.40–0.48)	<0.001 *	0.48 (0.43–0.52)	<0.001 *	
High PA	488/51,574 (1.0)	126,077	0.4	0.32 (0.29–0.35)	<0.001 *	0.35 (0.32–0.39)	<0.001 *	

CCI, Charlson comorbidity index; CIs, confidence intervals; DBP, diastolic blood pressure; fx, fracture; HR, hazard ratio; PA, physical activity; PY, person–year; SBP, systolic blood pressure. * Stratified Cox proportional hazard model, significance at <0.05 with Bonferroni correction (α = 0.05/3). ^1^ Stratified by gender, age, income within household unit, and region of residence. ^2^ Adjusted for total cholesterol, SBP, DBP, fasting blood glucose, obesity, smoking, alcohol consumption, and CCI score.

**Table 4:** HR (95% CIs) for distal radius fx in the PA groups with subgroup analyses according to age and gender.

Characteristics	No. of Distal Radius fx/ No. of Participants	Follow-Up Duration, PY	Incidence Rate, per 100 PY	Hazard Ratios for Distal Radius fx				*p* for Interaction
				Crude ^1^	*p*-Value	Adjusted ^1,2^	*p*-Value	
Total participants (*n* = 175,860)								
Low PA	8119/58,620 (13.9)	119,633	6.8	1		1		
Moderate PA	4229/58,620 (7.2)	132,804	3.2	0.48 (0.46–0.50)	<0.001 *	0.46 (0.45–0.48)	<0.001 *	
High PA	2991/58,620 (5.1)	137,923	2.2	0.33 (0.32–0.34)	<0.001 *	0.32 (0.30–0.33)	<0.001 *	
Age group								<0.001 *
Age < 65 years old (*n* = 38,367)								
Low PA	2812/12,789 (22.0)	23,162	12.1	1		1		
Moderate PA	1332/12,789 (10.4)	28,389	4.7	0.42 (0.40–0.44)	<0.001 *	0.41 (0.39–0.43)	<0.001 *	
High PA	898/12,789 (7.0)	30,308	3.0	0.27 (0.26–0.29)	<0.001 *	0.26 (0.25–0.28)	<0.001 *	
Age ≥ 65 years old (*n* = 137,493)								
Low PA	5307/45,831 (11.6)	96,471	5.5	1		1		
Moderate PA	2897/45,831 (6.3)	104,415	2.8	0.57 (0.54–0.60)	<0.001 *	0.55 (0.52–0.58)	<0.001 *	
High PA	2093/45,831 (4.6)	107,615	1.9	0.42 (0.39–0.45)	<0.001 *	0.40 (0.38–0.43)	<0.001 *	
Gender								0.008 *
Men (*n* = 21,138)								
Low PA	403/7046 (5.7)	16,895	2.4	1		1		
Moderate PA	282/7046 (4.0)	17,269	1.6	0.69 (0.59–0.81)	<0.001 *	0.66 (0.57–0.77)	<0.001 *	
High PA	212/7046 (3.0)	17,539	1.2	0.52 (0.44–0.61)	<0.001 *	0.49 (0.41–0.58)	<0.001 *	
Women (*n* = 154,722)								
Low PA	7716/51,574 (15.0)	102,738	7.5	1		1		
Moderate PA	3947/51,574 (7.7)	115,535	3.4	0.47 (0.45–0.49)	<0.001 *	0.45 (0.44–0.47)	<0.001 *	
High PA	2779/51,574 (5.4)	120,384	2.3	0.32 (0.31–0.33)	<0.001 *	0.31 (0.29–0.32)	<0.001 *	

CCI, Charlson comorbidity index; CIs, confidence intervals; DBP, diastolic blood pressure; fx, fracture; HR, hazard ratio; PA, physical activity; PY, person–year; SBP, systolic blood pressure. * Stratified Cox proportional hazard model, significance at <0.05 with Bonferroni correction (α = 0.05/3). ^1^ Stratified by gender, age, income within household unit, and region of residence. ^2^ Adjusted for total cholesterol, SBP, DBP, fasting blood glucose, obesity, smoking, alcohol consumption, and CCI score.

## Data Availability

Restrictions apply to the availability of these data. Data were obtained from the Korean National Health Insurance Sharing Service (NHISS) and are available at https://nhiss.nhis.or.kr (accessed on 25 January 2022) with the permission of the NHIS.

## Supplementary Materials

The following are available online at https://www.mdpi.com/article/10.3390/jpm12030491/s1, Table S1: Subgroup analyses of hazard ratio (95% confidence interval) for vertebral fx in the PA groups according to income, region of residence, obesity, smoking, alcohol consumption, total cholesterol, blood pressure, and fasting blood glucose, Table S2: Subgroup analyses of hazard ratio (95% confidence interval) for hip fx in the PA groups according to income, region of residence, obesity, smoking, alcohol consumption, total cholesterol, blood pressure, and fasting blood glucose, Table S3: Subgroup analyses of hazard ratio (95% confidence interval) for distal radius fx in the PA groups according to income, region of residence, obesity, smoking, alcohol consumption, total cholesterol, blood pressure, and fasting blood glucose, Table S4: Subgroup analyses of hazard ratios (95% confidence intervals) for each osteoporotic fracture in the PA groups according to previous PA level.
