# Supplementary material for: Physical Activity Is Associated with a Lower Risk of Osteoporotic Fractures in Osteoporosis: A Longitudinal Study
Source: J Pers Med. 2022 Mar 18;12(3):491. doi: 10.3390/jpm12030491 (PMC8949817; doi:10.3390/jpm12030491)
Supplement: Supplementary file 1 [file jpm-12-00491-s001.zip › Table S1(subgroup vertebral fx).pdf]

Table S1 Subgroup analyses of hazard ratio (95% confidence interval) for vertebral fx in the PA groups according to income, region of residence, obesity, smoking, alcohol consumption, total cholesterol, blood pressure, and fasting blood glucose

Characteristics	No. of vertebral fx/ No. of participants	Follow-up duration, PY	Incidence rate, per 100 PY	Hazard ratios for vertebral fx				P for interaction
				Crude[a]	P-value	Adjusted[ab]	P-value	
Income								0.093
Low income (n = 107,709)								
Low PA	7,776/35,903 (21.7)	66,844	11.6	1		1		
Moderate PA	3,695/35,903 (10.3)	79,642	4.6	0.42 (0.40-0.43)	<0.001*	0.42 (0.40-0.44)	<0.001*	
High PA	2,407/35,903 (6.7)	83,755	2.9	0.26 (0.25-0.27)	<0.001*	0.27 (0.25-0.28)	<0.001*	
High income (n = 68,151)								
Low PA	5,011/22,717 (22.1)	41,473	12.1	1		1		
Moderate PA	2,538/22,717 (11.2)	49,431	5.1	0.44 (0.42-0.46)	<0.001*	0.45 (0.42-0.47)	<0.001*	
High PA	1,635/22,717 (7.2)	52,345	3.1	0.27 (0.26-0.29)	<0.001*	0.28 (0.26-0.29)	<0.001*	
Region of residence								<0.001*
Urban (n = 76,569)								
Low PA	4,918/25,523 (19.3)	47,734	10.3	1		1		
Moderate PA	2,624/25,523 (10.3)	54,891	4.8	0.48 (0.46-0.50)	<0.001*	0.48 (0.46-0.51)	<0.001*	
High PA	1,683/25,523 (6.6)	58,032	2.9	0.30 (0.28-0.31)	<0.001*	0.30 (0.28-0.32)	<0.001*	

Rural (n = 99,291)								
Low PA	7,869/33,097 (23.8)	60,583	13.0	1		1		
Moderate PA	3,609/33,097 (10.9)	74,182	4.9	0.39 (0.38-0.41)	<0.001*	0.40 (0.38-0.41)	<0.001*	
High PA	2,359/33,097 (7.1)	78,068	3.0	0.25 (0.24-0.26)	<0.001*	0.25 (0.24-0.26)	<0.001*	
Obesity								0.093
Underweight (n = 5,981)								
Low PA	511/2,433 (21.0)	4,771	10.7	1		1		
Moderate PA	216/2,022 (10.7)	4,387	4.9	0.47 (0.40-0.56)	<0.001*	0.48 (0.41-0.56)	<0.001*	
High PA	122/1,526 (8.0)	3,471	3.5	0.34 (0.28-0.42)	<0.001*	0.37 (0.30-0.45)	<0.001*	
Normal weight (n = 66,256)								
Low PA	4,214/21,127 (20.0)	39,945	10.5	1		1		
Moderate PA	2,180/22,246 (9.8)	48,140	4.5	0.44 (0.42-0.47)	<0.001*	0.45 (0.43-0.47)	<0.001*	
High PA	1,385/22,883 (6.1)	51,859	2.7	0.27 (0.25-0.28)	<0.001*	0.27 (0.26-0.29)	<0.001*	
Overweight (n = 44,511)								
Low PA	3,083/13,857 (22.3)	25,559	12.1	1		1		
Moderate PA	1,577/15,003 (10.5)	33,184	4.8	0.41 (0.39-0.44)	<0.001*	0.41 (0.39-0.44)	<0.001*	
High PA	1,087/15,651 (7.0)	36,558	3.0	0.26 (0.25-0.28)	<0.001*	0.27 (0.25-0.29)	<0.001*	
Obese (n = 59,112)								
Low PA	4,979/21,203 (23.5)	38,042	13.1	1		1		

Moderate PA	2,260/19,349 (11.7)	43,362	5.2	0.42 (0.40-0.45)	<0.001*	0.42 (0.40-0.44)	<0.001*	
High PA	1,448/18,560 (7.8)	44,212	3.3	0.27 (0.26-0.29)	<0.001*	0.27 (0.25-0.29)	<0.001*	
Smoking status								<0.001*
Non-smoker (n = 158,863)								
Low PA	11,223/52,333 (21.5)	96,350	11.6	1		1		
Moderate PA	5,416/52,950 (10.2)	116,122	4.7	0.42 (0.41-0.43)	<0.001*	0.42 (0.41-0.44)	<0.001*	
High PA	3,509/53,580 (6.6)	124,019	2.8	0.26 (0.25-0.27)	<0.001*	0.26 (0.25-0.27)	<0.001*	
Past or current smoker (n = 16,997)								
Low PA	1,564/6,287 (24.9)	11,967	13.1	1		1		
Moderate PA	817/5,670 (14.4)	12,951	6.3	0.51 (0.47-0.56)	<0.001*	0.50 (0.46-0.54)	<0.001*	
High PA	533/5,040 (10.6)	12,081	4.4	0.36 (0.33-0.40)	<0.001*	0.36 (0.32-0.39)	<0.001*	
Alcohol consumption								0.475
< 1 time a week (n = 153,492)								
Low PA	11,283/51,745 (21.8)	95,563	11.8	1		1		
Moderate PA	5,377/51,022 (10.5)	112,229	4.8	0.43 (0.41-0.44)	<0.001*	0.43 (0.41-0.44)	<0.001*	
High PA	3,475/50,725 (6.9)	117,807	2.9	0.27 (0.26-0.28)	<0.001*	0.27 (0.26-0.28)	<0.001*	
≥ 1 time a week (n = 22,368)								
Low PA	1,504/6,875 (21.9)	12,754	11.8	1		1		

Moderate PA	856/7,598 (11.3)	16,844	5.1	0.45 (0.42-0.49)	<0.001*	0.45 (0.41-0.49)	<0.001*	
High PA	567/7,895 (7.2)	18,293	3.1	0.28 (0.25-0.31)	<0.001*	0.28 (0.25-0.30)	<0.001*	
Total cholesterol								0.836
< 200 mg/dL (n = 95,839)								
Low PA	6,832/31,663 (21.6)	57,127	12.0	1		1		
Moderate PA	3,376/31,943 (10.6)	68,190	5.0	0.43 (0.42-0.45)	<0.001*	0.44 (0.42-0.46)	<0.001*	
High PA	2,257/32,233 (7.0)	72,665	3.1	0.28 (0.26-0.29)	<0.001*	0.28 (0.27-0.30)	<0.001*	
≥ 200 to 240 < mg/dL (n = 55,466)								
Low PA	3,983/18,269 (21.8)	34,382	11.6	1		1		
Moderate PA	1,992/18,594 (10.7)	42,069	4.7	0.43 (0.41-0.45)	<0.001*	0.43 (0.41-0.45)	<0.001*	
High PA	1,223/18,603 (6.6)	44,525	2.7	0.25 (0.24-0.27)	<0.001*	0.26 (0.24-0.27)	<0.001*	
≥ 240 mg/dL (n = 24,555)								
Low PA	1,972/8,688 (22.7)	16,808	11.7	1		1		
Moderate PA	865/8,083 (10.7)	18,814	4.6	0.41 (0.38-0.45)	<0.001*	0.41 (0.38-0.45)	<0.001*	
High PA	562/7,784 (7.2)	18,910	3.0	0.27 (0.25-0.30)	<0.001*	0.27 (0.25-0.30)	<0.001*	
Blood pressure								0.006*
SBP <140 mmHg and DBP < 90 mmHg (n = 139,842)								
Low PA	9,678/45,582 (21.2)	82,969	11.7	1		1		

Moderate PA	4,733/46,931 (10.1)	101,607	4.7	0.42 (0.40-0.43)	<0.001*	0.42 (0.41-0.44)	<0.001*	
High PA	3,048/47,329 (6.4)	108,674	2.8	0.26 (0.25-0.27)	<0.001*	0.26 (0.25-0.27)	<0.001*	
SBP ≥ 140 mmHg or DBP ≥ 90 mmHg (n = 36,018)								
Low PA	3,109/13,038 (23.9)	25,348	12.3	1		1		
Moderate PA	1,500/11,689 (12.8)	27,466	5.5	0.47 (0.44-0.50)	<0.001*	0.47 (0.44-0.50)	<0.001*	
High PA	994/11,291 (8.8)	27,426	3.6	0.32 (0.29-0.34)	<0.001*	0.32 (0.29-0.34)	<0.001*	
Fasting blood glucose								0.140
< 100 mg/dL (n = 106,235)								
Low PA	7,623/34,571 (22.1)	64,143	11.9	1		1		
Moderate PA	3,793/35,946 (10.6)	80,669	4.7	0.42 (0.40-0.43)	<0.001*	0.42 (0.40-0.43)	<0.001*	
High PA	2,419/35,718 (6.8)	84,068	2.9	0.26 (0.25-0.27)	<0.001*	0.26 (0.25-0.27)	<0.001*	
≥ 100 mg/dL (n = 69,625)								
Low PA	5,164/24,049 (21.5)	44,174	11.7	1		1		
Moderate PA	2,440/22,674 (10.8)	48,404	5.0	0.45 (0.43-0.47)	<0.001*	0.46 (0.43-0.48)	<0.001*	
High PA	1,623/22,902 (7.1)	52,032	3.1	0.29 (0.27-0.30)	<0.001*	0.29 (0.27-0.31)	<0.001*	

Abbreviations: CCI, Charlson comorbidity index; DBP, diastolic blood pressure; fx, fx; PA, physical activity; PY, person-year; SBP, systolic blood pressure

* Cox proportional hazard model, Significance at P < 0.05 with Bonferroni correction

[a] Stratified by age, gender, income, and region of residence in subgroup analyses according to income and region of residence

[b] In subgroup analyses according to income and region of residence, the model was adjusted for total cholesterol, SBP, DBP, fasting blood glucose, obesity, smoking, alcohol consumption, and CCI score; in other subgroup analyses, the model was adjusted for above variable plus age, gender, income, and region of residence.
