# Supplementary material for: Physical Activity Is Associated with a Lower Risk of Osteoporotic Fractures in Osteoporosis: A Longitudinal Study
Source: J Pers Med. 2022 Mar 18;12(3):491. doi: 10.3390/jpm12030491 (PMC8949817; doi:10.3390/jpm12030491)
Supplement: Supplementary file 1 [file jpm-12-00491-s001.zip › Table S2(subgroup hip fx).pdf]

Table S2 Subgroup analyses of hazard ratio (95% confidence interval) for hip fx in the PA groups according to income, region of residence, obesity, smoking, alcohol consumption, total cholesterol, blood pressure, and fasting blood glucose

Characteristics	No. of hip fx/ No. of participants	Follow-up duration, PY	Incidence rate, per 100 PY	Hazard ratios for hip fx				P for interaction
				Crude[a]	P-value	Adjusted[ab]	P-value	
Income								0.776
Low income (n = 107,709)								
Low PA	1,176/35,903 (3.3)	86,111	1.4	1		1		
Moderate PA	567/35,903 (1.6)	87,770	0.6	0.47 (0.43-0.52)	<0.001*	0.51 (0.46-0.56)	<0.001*	
High PA	388/35,903 (1.1)	88,366	0.4	0.32 (0.29-0.36)	<0.001*	0.36 (0.32-0.40)	<0.001*	
High income (n = 68,151)								
Low PA	872/22,717 (3.8)	53,708	1.6	1		1		
Moderate PA	417/22,717 (1.8)	54,980	0.8	0.47 (0.42-0.52)	<0.001*	0.50 (0.45-0.57)	<0.001*	
High PA	299/22,717 (1.3)	55,341	0.5	0.33 (0.29-0.38)	<0.001*	0.37 (0.33-0.43)	<0.001*	
Region of residence								0.596
Urban (n = 76,569)								
Low PA	922/25,523 (3.6)	59,395	1.6	1		1		
Moderate PA	446/25,523 (1.8)	60,667	0.7	0.47 (0.42-0.53)	<0.001*	0.50 (0.45-0.56)	<0.001*	
High PA	323/25,523 (1.3)	61,130	0.5	0.34 (0.30-0.39)	<0.001*	0.37 (0.33-0.42)	<0.001*	

Rural (n = 99,291)								
Low PA	1,126/33,097 (3.4)	80,424	1.4	1		1		
Moderate PA	538/33,097 (1.6)	82,083	0.7	0.47 (0.42-0.52)	<0.001*	0.51 (0.46-0.56)	<0.001*	
High PA	364/33,097 (1.1)	82,577	0.4	0.32 (0.28-0.36)	<0.001*	0.36 (0.32-0.41)	<0.001*	
Obesity								0.272
Underweight (n = 5,981)								
Low PA	163/2,433 (6.7)	5,780	2.8	1		1		
Moderate PA	74/2,022 (3.7)	4,761	1.6	0.55 (0.42-0.73)	<0.001*	0.60 (0.45-0.79)	<0.001*	
High PA	36/1,526 (2.4)	3,691	1.0	0.35 (0.24-0.50)	<0.001*	0.42 (0.29-0.60)	<0.001*	
Normal weight (n = 66,256)								
Low PA	794/21,127 (3.8)	50,029	1.6	1		1		
Moderate PA	418/22,246 (1.9)	52,724	0.8	0.50 (0.44-0.56)	<0.001*	0.54 (0.48-0.60)	<0.001*	
High PA	278/22,883 (1.2)	54,288	0.5	0.32 (0.28-0.37)	<0.001*	0.37 (0.32-0.42)	<0.001*	
Overweight (n = 44,511)								
Low PA	462/13,857 (3.3)	33,368	1.4	1		1		
Moderate PA	215/15,003 (1.4)	36,789	0.6	0.42 (0.36-0.50)	<0.001*	0.45 (0.38-0.53)	<0.001*	
High PA	173/15,651 (1.1)	38,638	0.4	0.33 (0.27-0.39)	<0.001*	0.36 (0.30-0.43)	<0.001*	
Obese (n = 59,112)								
Low PA	629/21,203 (3.0)	50,642	1.2	1		1		

Moderate PA	277/19,349 (1.4)	48,476	0.6	0.46 (0.40-0.54)	<0.001*	0.49 (0.43-0.57)	<0.001*	
High PA	200/18,560 (1.1)	47,090	0.4	0.35 (0.30-0.41)	<0.001*	0.36 (0.31-0.43)	<0.001*	
Smoking status								0.006*
Non-smoker (n = 158,863)								
Low PA	1,628/52,333 (3.1)	124,566	1.3	1		1		
Moderate PA	751/52,950 (1.4)	128,194	0.6	0.45 (0.41-0.49)	<0.001*	0.48 (0.44-0.53)	<0.001*	
High PA	552/53,580 (1.0)	130,766	0.4	0.33 (0.30-0.36)	<0.001*	0.36 (0.32-0.39)	<0.001*	
Past or current smoker (n = 16,997)								
Low PA	420/6,287 (6.7)	15,253	2.8	1		1		
Moderate PA	233/5,670 (4.1)	14,556	1.6	0.59 (0.50-0.69)	<0.001*	0.60 (0.51-0.71)	<0.001*	
High PA	135/5,040 (2.7)	12,941	1.0	0.39 (0.32-0.47)	<0.001*	0.41 (0.34-0.50)	<0.001*	
Alcohol consumption								0.535
< 1 time a week (n = 153,492)								
Low PA	1,777/51,745 (3.4)	123,391	1.4	1		1		
Moderate PA	832/51,022 (1.6)	124,034	0.7	0.47 (0.43-0.51)	<0.001*	0.50 (0.46-0.55)	<0.001*	
High PA	595/50,725 (1.2)	124,362	0.5	0.34 (0.31-0.37)	<0.001*	0.38 (0.34-0.41)	<0.001*	
≥ 1 time a week (n = 22,368)								
Low PA	271/6,875 (3.9)	16,428	1.6	1		1		

Moderate PA	152/7,598 (2.0)	18,716	0.8	0.50 (0.41-0.61)	<0.001*	0.53 (0.43-0.65)	<0.001*	
High PA	92/7,895 (1.2)	19,345	0.5	0.29 (0.23-0.37)	<0.001*	0.32 (0.25-0.40)	<0.001*	
Total cholesterol								0.151
< 200 mg/dL (n = 95,839)								
Low PA	1,189/31,663 (3.8)	73,242	1.6	1		1		
Moderate PA	593/31,943 (1.9)	75,278	0.8	0.49 (0.44-0.54)	<0.001*	0.53 (0.48-0.59)	<0.001*	
High PA	445/32,233 (1.4)	76,742	0.6	0.36 (0.32-0.40)	<0.001*	0.41 (0.37-0.46)	<0.001*	
≥ 200 to 240 < mg/dL (n = 55,466)								
Low PA	590/18,269 (3.2)	44,524	1.3	1		1		
Moderate PA	279/18,594 (1.5)	46,613	0.6	0.45 (0.39-0.52)	<0.001*	0.47 (0.41-0.55)	<0.001*	
High PA	178/18,603 (1.0)	46,866	0.4	0.29 (0.25-0.34)	<0.001*	0.31 (0.26-0.37)	<0.001*	
≥ 240 mg/dL (n = 24,555)								
Low PA	269/8,688 (3.1)	22,053	1.2	1		1		
Moderate PA	112/8,083 (1.4)	20,859	0.5	0.44 (0.35-0.55)	<0.001*	0.48 (0.39-0.60)	<0.001*	
High PA	64/7,784 (0.8)	20,099	0.3	0.26 (0.20-0.35)	<0.001*	0.30 (0.23-0.40)	<0.001*	
Blood pressure								0.392
SBP <140 mmHg and DBP < 90 mmHg (n = 139,842)								
Low PA	1,478/45,582 (3.2)	106,876	1.4	1		1		

Moderate PA	732/46,931 (1.6)	111,944	0.7	0.47 (0.43-0.52)	<0.001*	0.51 (0.46-0.55)	<0.001*	
High PA	510/47,329 (1.1)	114,415	0.4	0.33 (0.29-0.36)	<0.001*	0.36 (0.33-0.40)	<0.001*	
SBP ≥ 140 mmHg or DBP ≥ 90 mmHg (n = 36,018)								
Low PA	570/13,038 (4.4)	32,943	1.7	1		1		
Moderate PA	252/11,689 (2.2)	30,806	0.8	0.48 (0.41-0.55)	<0.001*	0.51 (0.44-0.59)	<0.001*	
High PA	177/11,291 (1.6)	29,292	0.6	0.35 (0.30-0.42)	<0.001*	0.39 (0.32-0.46)	<0.001*	
Fasting blood glucose								0.004*
< 100 mg/dL (n = 106,235)								
Low PA	1,044/34,571 (3.0)	83,734	1.2	1		1		
Moderate PA	514/35,946 (1.4)	89,273	0.6	0.46 (0.42-0.52)	<0.001*	0.49 (0.44-0.54)	<0.001*	
High PA	350/35,718 (1.0)	88,892	0.4	0.32 (0.28-0.36)	<0.001*	0.35 (0.31-0.40)	<0.001*	
≥ 100 mg/dL (n = 69,625)								
Low PA	1,004/24,049 (4.2)	56,085	1.8	1		1		
Moderate PA	470/22,674 (2.1)	53,477	0.9	0.49 (0.44-0.55)	<0.001*	0.53 (0.47-0.59)	<0.001*	
High PA	337/22,902 (1.5)	54,815	0.6	0.35 (0.31-0.39)	<0.001*	0.39 (0.34-0.44)	<0.001*	

Abbreviations: CCI, Charlson comorbidity index; DBP, diastolic blood pressure; fx, fracture; PA, physical activity; PY, person-year; SBP, systolic blood pressure

* Cox proportional hazard model, Significance at $P < 0.05$ with Bonferroni correction

[a] Stratified by age, gender, income, and region of residence in subgroup analyses according to income and region of residence

[b] In subgroup analyses according to income and region of residence, the model was adjusted for total cholesterol, SBP, DBP, fasting blood glucose, obesity, smoking, alcohol consumption, and CCI score; in other subgroup analyses, the model was adjusted for above variable plus age, gender, income, and region of residence.
