# Supplementary material for: Physical Activity Is Associated with a Lower Risk of Osteoporotic Fractures in Osteoporosis: A Longitudinal Study
Source: J Pers Med. 2022 Mar 18;12(3):491. doi: 10.3390/jpm12030491 (PMC8949817; doi:10.3390/jpm12030491)
Supplement: Supplementary file 1 [file jpm-12-00491-s001.zip › Table S3(subgroup distal radius fx).pdf]

Table S3 Subgroup analyses of hazard ratio (95% confidence interval) for distal radius fx in the PA groups according to income, region of residence, obesity, smoking, alcohol consumption, total cholesterol, blood pressure, and fasting blood glucose

Characteristics	No. of distal radius fx/ No. of participants	Follow-up duration, PY	Incidence rate, per 100 PY	Hazard ratios for distal radius fx				P for interaction
				Crude[a]	P-value	Adjusted[ab]	P-value	
Income								0.008*
Low income (n = 107,709)								
Low PA	5,248/35,903 (14.6)	72,607	7.2	1		1		
Moderate PA	2,685/35,903 (7.5)	81,331	3.3	0.47 (0.44-0.49)	<0.001*	0.45 (0.43-0.47)	<0.001*	
High PA	1,881/35,903 (5.2)	84,593	2.2	0.32 (0.30-0.33)	<0.001*	0.30 (0.29-0.32)	<0.001*	
High income (n = 68,151)								
Low PA	2,871/22,717 (12.6)	47,026	6.1	1		1		
Moderate PA	1,544/22,717 (6.8)	51,473	3.0	0.50 (0.47-0.53)	<0.001*	0.49 (0.46-0.52)	<0.001*	
High PA	1,110/22,717 (4.9)	53,330	2.1	0.35 (0.33-0.38)	<0.001*	0.34 (0.32-0.36)	<0.001*	
Region of residence								<0.001*
Urban (n = 76,569)								
Low PA	3,409/25,523 (13.4)	50,972	6.7	1		1		
Moderate PA	1,929/25,523 (7.6)	56,060	3.4	0.53 (0.50-0.56)	<0.001*	0.51 (0.48-0.54)	<0.001*	
High PA	1,334/25,523 (5.2)	58,550	2.3	0.35 (0.33-0.38)	<0.001*	0.34 (0.32-0.36)	<0.001*	

Rural (n = 99,291)								
Low PA	4,710/33,097 (14.2)	68,661	6.9	1		1		
Moderate PA	2,300/33,097 (7.0)	76,744	3.0	0.44 (0.42-0.47)	<0.001*	0.43 (0.41-0.45)	<0.001*	
High PA	1,657/33,097 (5.0)	79,373	2.1	0.31 (0.30-0.33)	<0.001*	0.30 (0.28-0.32)	<0.001*	
Obesity								0.370
Underweight (n = 5,981)								
Low PA	254/2,433 (10.4)	5,410	4.7	1		1		
Moderate PA	131/2,022 (6.5)	4,588	2.9	0.61 (0.50-0.76)	<0.001*	0.59 (0.48-0.73)	<0.001*	
High PA	75/1,526 (4.9)	3,588	2.1	0.46 (0.35-0.59)	<0.001*	0.42 (0.32-0.54)	<0.001*	
Normal weight (n = 66,256)								
Low PA	3,126/21,127 (14.8)	42,370	7.4	1		1		
Moderate PA	1,653/22,246 (7.4)	48,937	3.4	0.47 (0.45-0.50)	<0.001*	0.45 (0.42-0.48)	<0.001*	
High PA	1,232/22,883 (5.4)	51,965	2.4	0.34 (0.31-0.36)	<0.001*	0.31 (0.29-0.33)	<0.001*	
Overweight (n = 44,511)								
Low PA	2,029/13,857 (14.6)	28,033	7.2	1		1		
Moderate PA	1,143/15,003 (7.6)	33,895	3.4	0.48 (0.45-0.52)	<0.001*	0.47 (0.43-0.50)	<0.001*	
High PA	775/15,651 (5.0)	37,061	2.1	0.31 (0.28-0.33)	<0.001*	0.29 (0.27-0.32)	<0.001*	
Obese (n = 59,112)								
Low PA	2,710/21,203 (12.8)	43,820	6.2	1		1		

Moderate PA	1,302/19,349 (6.7)	45,384	2.9	0.49 (0.45-0.52)	<0.001*	0.48 (0.44-0.51)	<0.001*	
High PA	909/18,560 (4.9)	45,309	2.0	0.35 (0.32-0.37)	<0.001*	0.34 (0.32-0.37)	<0.001*	
Smoking status								<0.001*
Non-smoker (n = 158,863)								
Low PA	7,594/52,333 (14.5)	104,838	7.2	1		1		
Moderate PA	3,919/52,950 (7.4)	118,587	3.3	0.47 (0.46-0.49)	<0.001*	0.45 (0.44-0.47)	<0.001*	
High PA	2,772/53,580 (5.2)	125,225	2.2	0.32 (0.31-0.34)	<0.001*	0.31 (0.29-0.32)	<0.001*	
Past or current smoker (n = 16,997)								
Low PA	525/6,287 (8.4)	14,795	3.5	1		1		
Moderate PA	310/5,670 (5.5)	14,217	2.2	0.63 (0.55-0.73)	<0.001*	0.64 (0.56-0.74)	<0.001*	
High PA	219/5,040 (4.4)	12,698	1.7	0.50 (0.43-0.59)	<0.001*	0.51 (0.44-0.60)	<0.001*	
Alcohol consumption								0.381
< 1 time a week (n = 153,492)								
Low PA	7,102/51,745 (13.7)	105,607	6.7	1		1		
Moderate PA	3,584/51,022 (7.0)	115,564	3.1	0.48 (0.46-0.50)	<0.001*	0.46 (0.44-0.48)	<0.001*	
High PA	2,558/50,725 (5.0)	119,400	2.1	0.34 (0.32-0.35)	<0.001*	0.32 (0.30-0.33)	<0.001*	
≥ 1 time a week (n = 22,368)								
Low PA	1,017/6,875 (14.8)	14,026	7.3	1		1		

Moderate PA	645/7,598 (8.5)	17,240	3.7	0.54 (0.49-0.59)	<0.001*	0.52 (0.47-0.57)	<0.001*	
High PA	433/7,895 (5.5)	18,523	2.3	0.34 (0.30-0.38)	<0.001*	0.33 (0.29-0.37)	<0.001*	
Total cholesterol								0.578
< 200 mg/dL (n = 95,839)								
Low PA	4,076/31,663 (12.9)	63,693	6.4	1		1		
Moderate PA	2,120/31,943 (6.6)	70,818	3.0	0.48 (0.46-0.51)	<0.001*	0.46 (0.43-0.48)	<0.001*	
High PA	1,527/32,233 (4.7)	74,060	2.1	0.34 (0.32-0.36)	<0.001*	0.31 (0.30-0.33)	<0.001*	
≥ 200 to 240 < mg/dL (n = 55,466)								
Low PA	2,706/18,269 (14.8)	37,482	7.2	1		1		
Moderate PA	1,448/18,594 (7.8)	42,820	3.4	0.49 (0.46-0.52)	<0.001*	0.47 (0.44-0.50)	<0.001*	
High PA	1,039/18,603 (5.6)	44,775	2.3	0.34 (0.32-0.37)	<0.001*	0.32 (0.30-0.35)	<0.001*	
≥ 240 mg/dL (n = 24,555)								
Low PA	1,337/8,688 (15.4)	18,458	7.2	1		1		
Moderate PA	661/8,083 (8.2)	19,166	3.4	0.49 (0.45-0.54)	<0.001*	0.48 (0.43-0.52)	<0.001*	
High PA	425/7,784 (5.5)	19,088	2.2	0.32 (0.29-0.36)	<0.001*	0.31 (0.27-0.34)	<0.001*	
Blood pressure								0.052
SBP <140 mmHg and DBP < 90 mmHg (n = 139,842)								
Low PA	6,525/45,582 (14.3)	90,221	7.2	1		1		

Moderate PA	3,426/46,931 (7.3)	103,673	3.3	0.47 (0.46-0.49)	<0.001*	0.45 (0.44-0.47)	<0.001*	
High PA	2,432/47,329 (5.1)	109,727	2.2	0.33 (0.31-0.34)	<0.001*	0.31 (0.29-0.32)	<0.001*	
SBP ≥ 140 mmHg or DBP ≥ 90 mmHg (n = 36,018)								
Low PA	1,594/13,038 (12.2)	29,412	5.4	1		1		
Moderate PA	803/11,689 (6.9)	29,131	2.8	0.53 (0.48-0.57)	<0.001*	0.52 (0.47-0.56)	<0.001*	
High PA	559/11,291 (5.0)	28,196	2.0	0.38 (0.35-0.42)	<0.001*	0.37 (0.34-0.41)	<0.001*	
Fasting blood glucose								0.333
< 100 mg/dL (n = 106,235)								
Low PA	5,122/34,571 (14.8)	69,997	7.3	1		1		
Moderate PA	2,678/35,946 (7.5)	82,556	3.2	0.46 (0.44-0.49)	<0.001*	0.44 (0.42-0.47)	<0.001*	
High PA	1,905/35,718 (5.3)	85,063	2.2	0.32 (0.31-0.34)	<0.001*	0.30 (0.29-0.32)	<0.001*	
≥ 100 mg/dL (n = 69,625)								
Low PA	2,997/24,049 (12.5)	49,636	6.0	1		1		
Moderate PA	1,551/22,674 (6.8)	50,248	3.1	0.52 (0.49-0.56)	<0.001*	0.50 (0.47-0.54)	<0.001*	
High PA	1,086/22,902 (4.7)	52,860	2.1	0.36 (0.33-0.38)	<0.001*	0.34 (0.32-0.36)	<0.001*	

Abbreviations: CCI, Charlson comorbidity index; DBP, diastolic blood pressure; fx, fracture; PA, physical activity; PY, person-year; SBP, systolic blood pressure

* Cox proportional hazard model, Significance at $P < 0.05$ with Bonferroni correction

[a] Stratified by age, gender, income, and region of residence in subgroup analyses according to income and region of residence

[b] In subgroup analyses according to income and region of residence, the model was adjusted for total cholesterol, SBP, DBP, fasting blood glucose, obesity, smoking, alcohol consumption, and CCI score; in other subgroup analyses, the model was adjusted for above variable plus age, gender, income, and region of residence.
