# Supplementary material for: Physical Activity Is Associated with a Lower Risk of Osteoporotic Fractures in Osteoporosis: A Longitudinal Study
Source: J Pers Med. 2022 Mar 18;12(3):491. doi: 10.3390/jpm12030491 (PMC8949817; doi:10.3390/jpm12030491)
Supplement: Supplementary file 1 [file jpm-12-00491-s001.zip › Table S4(additional subgroup).pdf]

Table S4 Subgroup analyses of hazard ratios (95% confidence intervals) for each osteoporotic fracture in the PA groups according to previous PA level

Characteristics	No. of fx/ No. of participants	Hazard ratios for each fx			
		Crude	P-value	Adjusted[a]	P-value
Hazard ratios for vertebral fx					
Low PA previously (n = 60,995)					
Low PA	5,231/25,477 (20.5)	1		1	
Moderate PA	1,872/20,304 (9.2)	0.37 (0.35-0.39)	<0.001*	0.37 (0.35-0.39)	<0.001*
High PA	952/15,214 (6.3)	0.24 (0.22-0.26)	<0.001*	0.24 (0.23-0.26)	<0.001*
Moderate PA previously (n = 45,618)					
Low PA	2,218/10,942 (20.3)	1		1	
Moderate PA	1,479/16,420 (9.0)	0.40 (0.37-0.43)	<0.001*	0.40 (0.38-0.43)	<0.001*
High PA	1,005/18,256 (5.5)	0.23 (0.21-0.24)	<0.001*	0.23 (0.22-0.25)	<0.001*
High PA previously (n = 10,488)					
Low PA	302/1,426 (21.2)	1		1	
Moderate PA	242/2,607 (9.3)	0.44 (0.37-0.52)	<0.001*	0.45 (0.38-0.54)	<0.001*
High PA	367/6,455 (5.7)	0.25 (0.21-0.29)	<0.001*	0.27 (0.23-0.31)	<0.001*
Non-information of previous PA (n = 58,759)					
Low PA	5,036/20,775 (24.2)	1		1	
Moderate PA	2,640/19,289 (13.7)	0.50 (0.48-0.53)	<0.001*	0.50 (0.48-0.52)	<0.001*
High PA	1,718/18,695 (9.2)	0.33 (0.31-0.35)	<0.001*	0.33 (0.31-0.34)	<0.001*
Hazard ratios for hip fx					
Low PA previously (n = 60,995)					
Low PA	761/25,477 (3.0)	1		1	
Moderate PA	236/20,304 (1.2)	0.36 (0.31-0.42)	<0.001*	0.39 (0.34-0.46)	<0.001*
High PA	156/15,214 (1.0)	0.31 (0.26-0.37)	<0.001*	0.36 (0.30-0.43)	<0.001*
Moderate PA previously (n = 45,618)					
Low PA	321/10,942 (2.9)	1		1	

Moderate PA	238/16,420 (1.5)	0.50 (0.42-0.59)	<0.001*	0.54 (0.46-0.64)	<0.001*
High PA	165/18,256 (0.9)	0.30 (0.25-0.36)	<0.001*	0.35 (0.29-0.42)	<0.001*
High PA previously (n = 10,488)					
Low PA	49/1,426 (3.4)	1		1	
Moderate PA	40/2,607 (1.5)	0.50 (0.33-0.75)	0.001	0.54 (0.35-0.82)	0.004
High PA	61/6,455 (1.0)	0.29 (0.20-0.43)	<0.001*	0.35 (0.24-0.51)	<0.001*
Non-information of previous PA (n = 58,759)					
Low PA	917/20,775 (4.4)	1		1	
Moderate PA	470/19,289 (2.4)	0.54 (0.48-0.60)	<0.001*	0.57 (0.51-0.64)	<0.001*
High PA	305/18,695 (1.6)	0.36 (0.32-0.41)	<0.001*	0.39 (0.34-0.45)	<0.001*
Hazard ratios for distal radius fx					
Low PA previously (n = 60,995)					
Low PA	3,334/25,477 (13.1)	1		1	
Moderate PA	1,260/20,304 (6.2)	0.42 (0.39-0.44)	<0.001*	0.39 (0.37-0.42)	<0.001*
High PA	659/15,214 (4.3)	0.28 (0.26-0.31)	<0.001*	0.26 (0.24-0.28)	<0.001*
Moderate PA previously (n = 45,618)					
Low PA	1,535/10,942 (14.0)	1		1	
Moderate PA	1,063/16,420 (6.5)	0.44 (0.40-0.47)	<0.001*	0.41 (0.38-0.44)	<0.001*
High PA	876/18,256 (4.8)	0.31 (0.28-0.33)	<0.001*	0.28 (0.25-0.30)	<0.001*
High PA previously (n = 10,488)					
Low PA	190/1,426 (13.3)	1		1	
Moderate PA	194/2,607 (7.4)	0.58 (0.48-0.71)	<0.001*	0.55 (0.45-0.67)	<0.001*
High PA	321/6,455 (5.0)	0.37 (0.31-0.44)	<0.001*	0.33 (0.28-0.40)	<0.001*
Non-information of previous PA (n = 58,759)					
Low PA	3,060/20,775 (14.7)	1		1	
Moderate PA	1,712/19,289 (8.9)	0.57 (0.54-0.61)	<0.001*	0.56 (0.52-0.59)	<0.001*
High PA	1,135/18,695 (6.1)	0.39 (0.36-0.41)	<0.001*	0.37 (0.35-0.40)	<0.001*

Abbreviations: CCI, Charlson comorbidity index; DBP, diastolic blood pressure; fx, fracture; SBP, systolic blood pressure

* Cox proportional hazard model, Significance at $P < 0.05$ with Bonferroni correction

[a] The model was adjusted for age, gender, income, region of residence, total cholesterol, SBP, DBP, fasting blood glucose, obesity, smoking, alcohol consumption, and CCI score
